# Matrix Metalloproteinases Retain Soluble FasL-mediated Resistance to Cell Death in Fibrotic-Lung Myofibroblasts

**DOI:** 10.3390/cells9020411

**Published:** 2020-02-11

**Authors:** David Nareznoi, Jenya Konikov-Rozenman, Dmytro Petukhov, Raphael Breuer, Shulamit B. Wallach-Dayan

**Affiliations:** 1 Lung Cellular and Molecular Biology Laboratory, Institute of Pulmonary Medicine, Hadassah Medical Research Center, PO Box 12000, Kiryat Hadassah, Jerusalem 91120, Israel; david.nareznoy@gmail.com (D.N.); jenyak86@gmail.com (J.K.-R.); petukhov@hadassah.org.il (D.P.); raffibreuer@gmail.com (R.B.); 2 Department of Pathology and Laboratory Medicine, 670 Albany St, 4th Floor, Boston University School of Medicine, Boston, MA 02118, USA

**Keywords:** pulmonary fibrosis, lung myofibroblasts, matrix metalloproteinase (MMP), soluble FasL (sFasL), cell death

## Abstract

A prominent feature of obstructed tissue regeneration following injury in general, and fibrotic lung tissue in particular, is fibroblast proliferation and accumulation. The Fas/FasL apoptotic pathway has been shown to be involved in human idiopathic pulmonary fibrosis (IPF) and bleomycin-induced lung fibrosis in rodents. We previously showed that in normal injury repair, myofibroblasts’ accumulation is followed by their decline by FasL^+^ T cell-induced cell death. In pathological lung fibrosis, myofibroblasts resist cell death and accumulate. Like other members of the tumor necrosis factor (TNF) family, membrane-bound FasL can be cleaved from the cell surface to generate a soluble form (sFasL). Metalloproteinases (MMPs) are known to convert the membrane-bound form of FasL to sFasL. MMP-7 knockout (KO) mice were shown to be protected from bleomycin (BLM)-induced lung fibrosis. In this study, we detected increased levels of sFasL in their blood serum, as in the lungs of patients with IPF, and IPF-lung myofibroblast culture medium. In this study, using an MMP-inhibitor, we showed that sFasL is decreased in cultures of IPF-lung myofibroblasts and BLM-treated lung myofibroblasts, and in the blood serum of MMP-7KO mice. Moreover, resistant fibrotic-lung myofibroblasts, from the lungs of humans with IPF and of BLM-treated mice, became susceptible to T-cell induced cell death in a co-culture following MMP-inhibition- vs. control-treatment or BLM-treated MMP-7KO vs. wild-type mice, respectively. sFasL may be an unrecognized mechanism for MMP-7-mediated decreased tissue regeneration following injury and the evolution of lung fibrosis.

## 1. Introduction

Idiopathic pulmonary fibrosis (IPF) is a non-neoplastic pulmonary disease characterized by the lack of tissue regeneration and formation of scar tissue within the lungs, in the absence of any known provocation [1,2]. The median survival of patients with IPF is approximately 3 years from the time of diagnosis [3]. It is characterized by alveolar epithelial injury, inflammatory cell accumulation, myofibroblast hyperplasia, the deposition of extracellular matrix and scars. Myofibroblasts are a source of collagen [4,5], cytokines, chemokines, and profibrogenic growth factors [6,7,8].

T lymphocyte-mediated cell death constitutes an important component of specific effector mechanisms in the immune surveillance against unwanted cells [9]. T lymphocytes have been found in close proximity to interstitial myofibroblasts during pulmonary fibrosis [10], and a direct relationship between the presence of T cells and myofibroblast apoptosis has been reported [11]. T cell-myofibroblast interaction has been shown to play a role in a number of physiological as well as pathological states, including the regulation of wound healing [12,13].

Fas ligand (FasL or CD95L) is a type-II transmembrane protein that belongs to the tumor necrosis factor (TNF) family. It binds with its receptor, Fas [14,15], and induces apoptosis [16,17].

Like other members of the TNF family, membrane-bound FasL (mFasL) can be cleaved from the cell surface to generate a 26-kD soluble form (sFasL). The role of sFasL has been less clear. Even though sFasL does not efficiently induce apoptosis, it has been shown to bind Fas and specifically block the apoptotic activity of membrane-bound FasL [18,19]. Significantly, rather than inducing chemotaxis, the natural cleavage product actually opposed the activity of mFasL and protected cells from neutrophil-effector mechanisms [20]. Matrix metalloproteinases (MMPs) are known to convert mFasL to sFasL [21,22]. MMPs are essential for extracellular matrix remodeling, wound healing, and angiogenesis, and have been implicated in the pathogenesis of IPF [23,24]. The role of MMP-7 in the progression of interstitial lung diseases, and of IPF in particular, has been firmly established by numerous studies [25,26,27,28,29]. MMP-7 expression is elevated in both human IPF and murine models of fibrosis, while MMP-7 knockout (KO) mice have attenuated fibrotic reactions [23,24]. MMP-7 is known to be produced by both alveolar epithelial cells and myofibroblasts, and it plays a role in various processes associated with the initiation and progression of pulmonary fibrosis, such as epithelial-mesenchymal transition, extracellular matrix degradation, aberrant matrix repair, and tissue remodeling [26,28,30,31]. As sFasL demonstrates the capacity to promote fibroblast survival and apoptotic resistance [32], and MMP-7 is an important FasL shedder [33], the involvement of MMP-7 in cell survival may be conjectured. There are indications that MMP-7 may play a part in promoting cell survival and resistance to apoptosis [34], for example via the cleavage of osteopontin [35], which in turn may be upregulated via RUNX2 expression [36]. Nevertheless, the multifaceted role of MMP-7 in pulmonary fibrosis and fibroblast survival via sFasL still requires further study. Here, we assessed an unrecognized relationship of MMP-7 with fibrotic-lung myofibroblast resistance to cell death via sFasL cleavage.

## 2. Material and Methods

**Animals.** C57BL/6 mice, male, 11–12 weeks old (Harlan Sprague Dawley, Indianapolis, IN, USA) and B6.129-*Mmp7^tm1Lmm^*/J mice of the same sex and age (Jackson Laboratory, Bar Harbor, MN, USA), were used. All experiments conform to the relevant regulatory standards and were approved by the Institutional Animal Care and Use Committee of the Hadassah-Hebrew University Medical Center (Number: MD-11-12314-4). Mice were maintained under specific pathogen-free conditions with adherence to institutional guidelines for the care and use of laboratory animals. 

**Bleomycin lung injury.** Oropharyngeal aspiration of bleomycin (BLM) was performed, as previously detailed [37]. 

**Human lung fibroblasts, T-cell lines, and mouse myofibroblasts from fibrotic-mouse lungs and culture**. IPF lung myofibroblast cell line CCL-191, normal lung fibroblast cell line CCL-151, and Jurkat T-cell line were obtained from American Type Culture Collection-ATCC (Manassas, VA, USA). The cells were cultured in Dulbecco’s modified Eagle medium (DMEM) supplemented with 10% fetal bovine serum (FBS) at 37 °C in a 5% humidified CO_2_ incubator. The cells were passaged two times per week at up to 10 passages.

Myofibroblast isolation from fibrotic mouse lungs has been described by us in detail previously [37,38]. As detailed, the myofibroblast profile was confirmed by the elevated expression of α-smooth muscle actin [39]. The cells were cultured in RPMI 1640 Medium supplemented with 10% FBS. 

**Lung Myofibroblast with Jurkat T cell Co-Cultures and Cell-death.** Normal lung fibroblasts or IPF lung myofibroblasts were co-cultured with Jurkat T cells for 48 h in DMEM with 10% FBS, to determine the induction of cell-cell death. Lung fibroblasts were plated at a density of 3 × 10^5^ in 6-well tissue culture plates. T cells were added to normal fibroblasts and IPF lung myofibroblasts after 24 h of batimastat treatment. Following the co-culture, myofibroblasts were exposed to trypan blue dye, and the detection of viable cells was performed by trypan blue exclusion as we described previously [37,38,40].

**Immunoblotting of FasL** was conducted as we previously detailed [41]. Briefly, equal amounts of normal and IPF lung myofibroblasts culture medium (5 mL), or of normal or bleomycin-treated wild type and MMP7KO mice blood serum (10 µL), were sampled. Soluble proteins were precipitated from culture medium or blood serum by ultracentrifugation in a TL-100 New Ultracentrifuge at 100,000× *g* for 2 h at 4 °C. Equal amounts of protein were loaded on 12.5% sodium dodecyl sulfate-polyacrylamide (SDS-PAGE) gels and electrophoretically transferred to membranes. The blotted membrane was blocked in 1% casein PBS-buffered solution (Bio-Rad, Hercules, CA, USA) for 1 h at room temperature. FasL was detected using NOK-1 anti-sFasL antibody incubation overnight at 4 °C (clone 101626, R&D, Minneapolis, MN, USA), followed by incubation for 1 h at room temperature with horseradish peroxidase-conjugated (HRP) antiserum in 1% casein-PBS solution. The membrane was developed with Immobilon Western HRP substrate solution (Millipore, Burlington, MA, USA). The imaging was performed with ChemiDoc XRS+ transilluminator, and ImageLab 4.0 software was used for the densitometry analysis (Bio-Rad Laboratories, Hercules, CA, USA). A quantitative analysis was performed and summarized graphically as we previously detailed [42]. Soluble FasL secreted by fibroblasts into culture medium was normalized to GAPDH levels detected in cells lysates from the same cell culture following the standard process using anti-GAPDH antibody (Santa Cruz Biotechnology, Dallas, TX, USA) [41]. Membrane FasL (mFasL) was detected in same cell-lysates using anti-sFasL antibody (clone 101626, R&D, Minneapolis, MN, USA).

**Data analysis and statistics.** The Kruskall-Wallis test was applied to compare variables measured at different time intervals or following different treatments. The Mann-Whitney test with the Bonferroni correction was used to test for statistical significance. A two-way ANOVA was used to assess the time and treatment effects and interactions. The data are represented as the mean with standard deviation; the number of experiments (n) is indicated for each experimental series, where applicable.

## 3. Results

**Figure 1:**
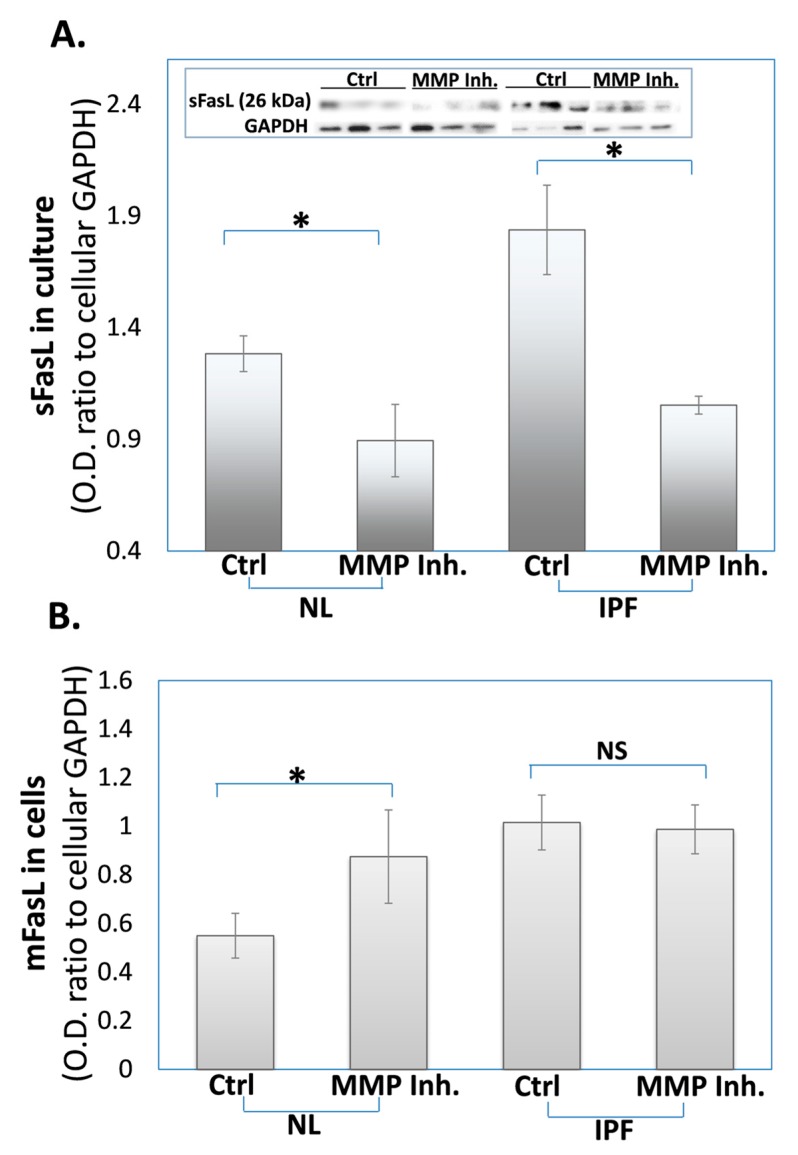
The decreased soluble and increased membrane FasL levels in fibrotic-(IPF) and normal (NL)-lung myofibroblasts, following exposure to the batimastat MMP inhibitor. Western blot of: (**A**) sFasL in culture medium and (**B**) mFasL, of fibroblast cell lines (3 × 10^5^) of fibrotic-lung/ATCC191 (IPF)- or normal/ATCC151 (NL)-lungs; Graphical presentation and blots (insert) with optical density ratios normalized to fibroblasts GAPDH after treatment with control-vehicle (0.1% DMSO) or batimastat (24 h, 10 µM). Mean ± standard deviation; *n* = 4; * *p* < 0.05, NS—*p* > 0.05.

IPF-lung myofibroblasts release less FasL into their culture medium (sFasL), with a concomitant increase in membrane levels (mFasL), following exposure to an MMP inhibitor (batimastat). It has been previously shown by others, in cells from tumors and tissues [18,43,44], that mFasL can be cleaved by MMPs, resulting in sFasL release to the cell milieu. We aimed to assess our hypothesis that MMPs are responsible for the increased sFasL levels that we had previously detected in the culture medium of IPF-lung myofibroblasts [38]. To this end, IPF-lung myofibroblasts were treated with a pan-MMP inhibitor, batimastat (10 μM), or with a control-vehicle (0.1% dimethyl sulfoxide (DMSO)), for 24 h., and the levels of sFasL from the culture medium or mFasL from the cultured cells were assessed by a Western blot analysis as detailed in the methods. When compared to the controls, vehicle-treated IPF, or normal (NL) cells, the sFasL levels decreased in the culture of IPF-lung myofibroblasts following treatment with the MMP inhibitor, from an OD of 1.8 ± 0.2SD to only 1.1 ± 0.1SD, compared with a decrease from 1.3 ± 0.1SD to 0.9 ± 0.2SD OD in the normal cells (Figure 1A; sFasL in IPF or NL cells +Ctrl or +MMP inhibitor). The mFasL levels were also assessed in cultured cell-lysates and were found to be higher following the MMP inhibitor treatment of normal-lung myofibroblasts, from an OD of 0.5 ± 0.1SD to 0.9 ± 0.2SD, but with comparable levels in the IPF cells, which were relatively higher in the baseline levels 0.9–1 OD (Figure 1B; mFasL in IPF or NL cells +Ctrl or +MMP inhibitor). To equalize the initial protein quantities, GAPDH was detected in the cultured cell-lysates, and OD ratios of sFasL and mFasL to GAPDH were calculated (see graphical presentation and inserts). 

**The MMP inhibitor reverses IPF-lung myofibroblast resistance to cell death.** It has been previously reported by us [42] and others [45] that normal lung myofibroblasts, but not those from fibrotic lungs in bleomycin-treated mice and in humans with IPF, are susceptible to apoptosis induced by Fas agonists and immune T cells. mFasL can also induce cell death [18,45], and sFasL (sFasL) contributes to the resistance to Fas- and immune cell-induced death [32]. Having shown [42], that IPF-media can induce resistance to cell death in normal lung myofibroblasts as in IPF-cells due to the elevated levels of sFasL in the medium, we blocked the cleavage of FasL that was initiated by MMP using MMP inhibitor batimastat (10 μM, for 24 h), compared to the vehicle (DMSO). 

**Figure 2:**
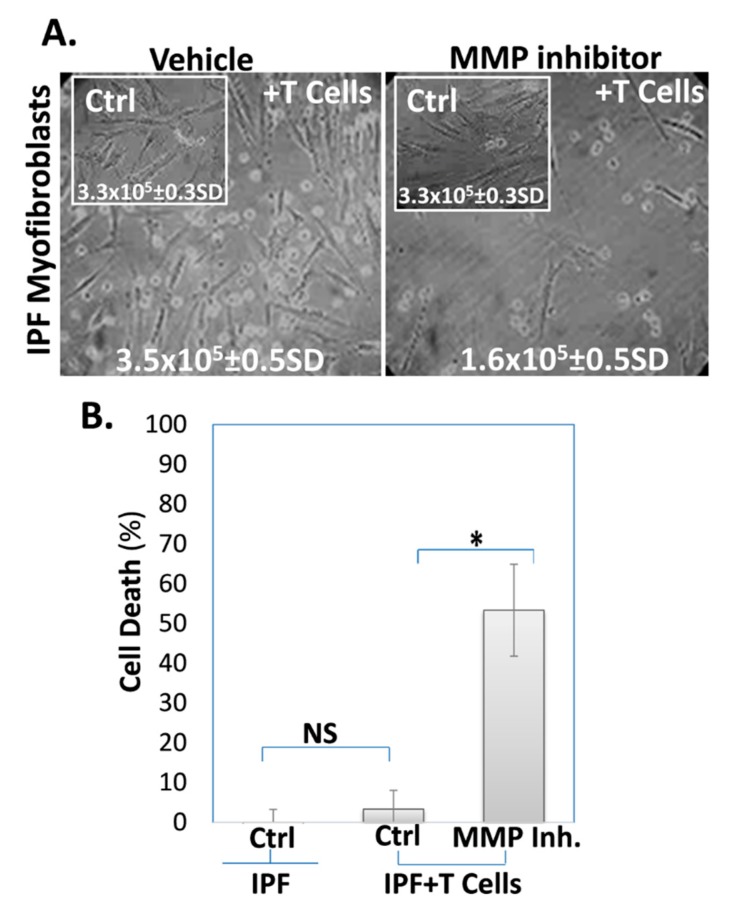
The MMP inhibitor (batimastat) decreases IPF-lung myofibroblast’ resistance to T cell-induced cell death. **(A)** Light microscope images with trypan blue exclusion (inserted numbers); and (**B**) Graphical presentation of the cell death percentage defined by trypan blue exclusion. Comparisons were made between IPF-lung myofibroblasts (ATCC-191 cell-line) cultured alone (inserts-Ctrl) and IPF-lung myofibroblasts co-cultured with T cells (1 × 10^6^, 48 h), following being treated with vehicle (0.1% DMSO) or MMP-inhibitor (batimastat 10 µM, 24 h), (vehicle or MMP inhibitor, +T cells). The percentage of dead cells to total cell count in each sample was analyzed as the mean ± standard deviation; *n* = 3; * *p* < 0.05, NS *p* > 0.05.

Our results clearly show (Figure 2) that IPF-lung myofibroblasts do not change their density, as can be seen in Figure 2A (microscope images, IPF + T cells vs. control IPF cells (insert) and cell count of approx. 3.5 × 10^5^ and 3.3 × 10^5^ (Figure 2A, inserted numbers, IPF + T cells vs. control IPF cells)), after co-culture with T cells. However, after being exposed to the pan-MMP inhibitor (batimastat), half of IPF-lung myofibroblasts die in the co-culture (Figure 1A; images of IPF + T cells+ MMP inhibitor vs. Ctrl), and their cell count is markedly decreased from 3.5 × 10^5^ to only 1.6 × 10^5^ (Figure 1A inserted numbers and Figure 2B; graphical presentation, IPF + T cells vs. control IPF cells). The graphical presentation of the trypan blue-exclusion shows that, whereas IPF cells resist cell death which reaches a maximum of only 2–5% dead cells (Figure 2B; IPF VS. IPF + T cells), IPF cells treated with the MMP inhibitor increase their susceptibility to cell death to almost 60% (Figure 2B; IPF + T cells + MMP inhibitor vs. Ctrl). These results indicate that when exposed to batimastat, which annuls the release of sFasL into the culture medium (Figure 1A), IPF-lung myofibroblasts lose their resistance to cell death compared to non-treated IPF cells.

**Figure 3:**
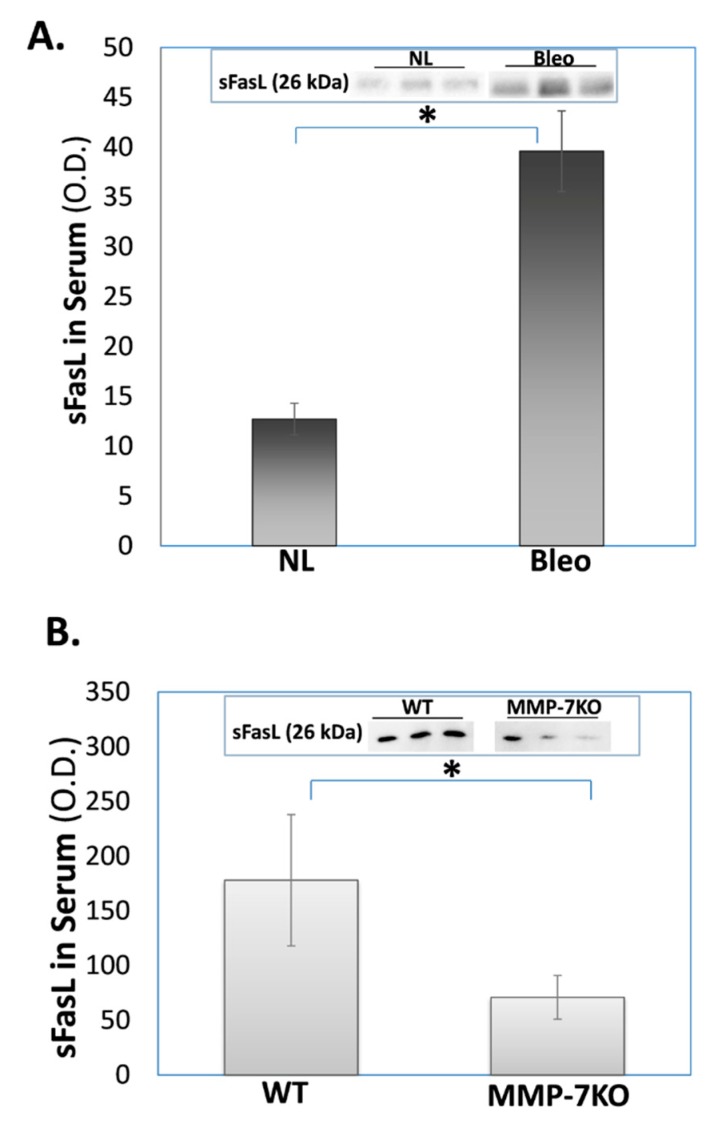
sFasL is increased in the blood serum of wild type (WT), but not of MMP-7 knock-out (KO) bleomycin-treated, mice. The graphical presentation and inserts of WB with the OD analysis of sFasL assessed from equal volumes of blood serum samples (10 μL) of day 14 of (**A**) bleomycin (Bleo) vs. saline (NL)-treated WT mice, and (**B**) bleomycin-treated WT vs. MMP-7 KO mice. Mean ± standard deviation; *n* = 4; *p < 0.05.

The sFasL levels are increased in the blood serum of bleomycin-, compared to normal saline- treated wild type (WT) mice, whereas sFasL returns to normal levels in MMP-7 knockout (KO) mice compared to their WT counterparts. Patients with IPF manifested increased levels of sFasL in their blood circulation [46,47,48] and in fluids obtained via broncheoalveolar lavage (BAL) [48], which correlated with disease activity [46]. We initially confirmed that, as in the serum of humans with IPF, bleomycin-treated WT mice manifested increased sFasL levels in their blood stream (Figure 3A). As in result 1, we evaluated the sFasL levels in the blood serum of mice with bleomycin-induced lung fibrosis (day 14 post Bleo), compared to normal-saline treated lungs. Following the ultracentrifugation of the blood serum, sFasL levels were determined in WB using specific NOK-1 anti sFasL mAb (see methods). The levels were significantly higher in the blood of mice with bleomycin (Bleo)-induced fibrosis (Figure 3A, graphical presentation and insert), with 3-fold increases of 39 ± 4SD OD following bleomycin, vs. 13 ± 2SD OD in the serum of saline (NL)-treated mice lungs. 

Our objective was to assess the critical role that MMPs (and MMP-7 in particular) play on sFasL upregulation in the blood serum of mice. To this end, we assessed changes in the sFasL levels in MMP^+^ vs. MMP^−^ blood serum isolated from WT and MMP-7 KO mice, respectively. A 2.5-fold decrease was detected in WB, from an average of 178 ± 40SD OD to 71 ± 9SD OD in sFasL levels at day 14 of the bleomycin instillation in cells from MMP-7KO mice compared to those from their WT counterparts (Figure 3B, graphical presentation and insert). 

**Figure 4:**
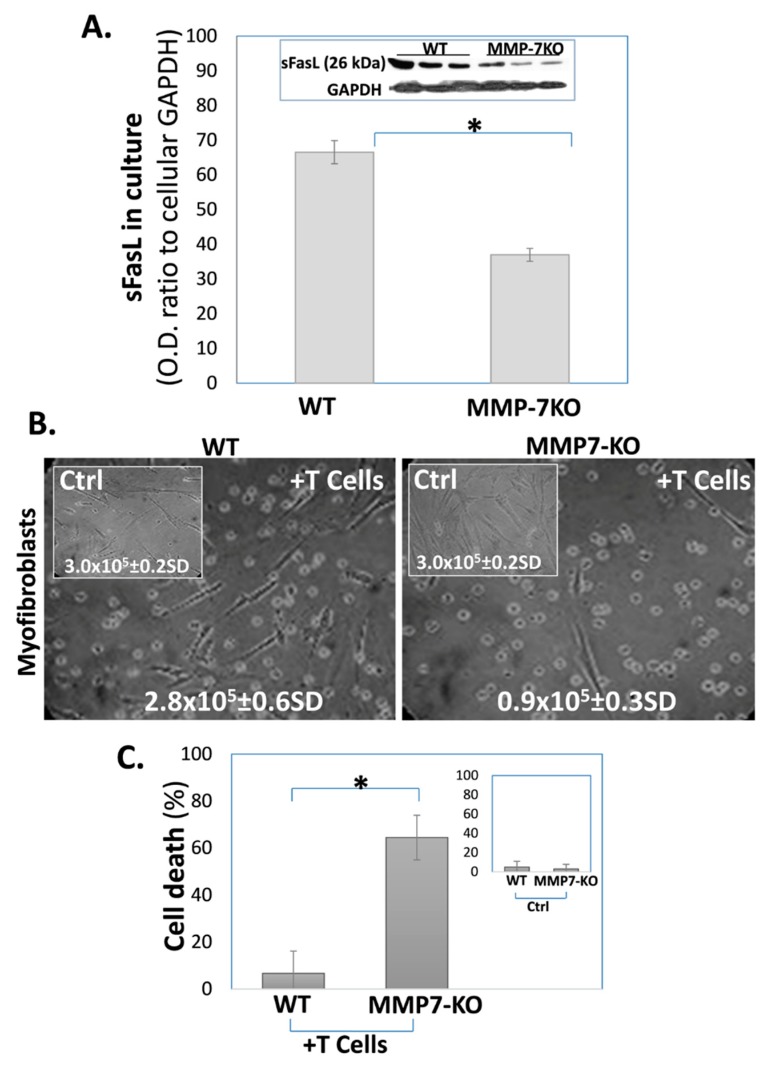
The decreased levels of sFasL in the culture medium of MMP-7 KO lung myofibroblasts increase their susceptibility to cell death when further co-cultured with T cells. (**A**) Western blot of sFasL in culture medium, of lung fibroblasts (3 × 10^5^), isolated from bleomycin-treated lungs (day 14) of WT vs. MMP-7 KO mice; Graphical presentation and blots (insert) with optical density ratios normalized to fibroblasts’ GAPDH. Mean ± standard deviation; *n* = 3; * *p* < 0.05. (**B**) Light microscope images with trypan blue exclusion (inserted numbers); and (**C**) Graphical presentation of the cell death percentage defined by trypan blue exclusion. Comparisons were made between myofibroblasts from lungs of bleomycin-treated wild type (WT) vs. MMP7 knockout (MMP-7 KO) cultured alone (inserts-Ctrl) and co-cultured with T cells (1 × 10^6^, 48 h), (WT or MMP7-KO, +T cells). Mean ± standard deviation; *n* = 3; *p < 0.05.

Fibroblasts from MMP-7 KO mice, as opposed to those from WT counterparts, secrete lower sFasL levels and, following co-culture with T-cells, undergo cell death. In this experiment, we aimed to evaluate the role of MMPs in the sFasL secretion into cell culture medium by fibroblasts. In order to do this, we assessed the changes in the sFasL levels in the cell culture of myofibroblasts isolated from the lungs of bleomycin-treated WT and MMP-7 KO mice. As expected, lung myofibroblasts from MMP-7 KO mice released much less sFasL into the culture medium when compared to those from WT mice, from an average OD of 67 ± 5SD to 37 ± 3SD, respectively (Figure 4A).

Following the assessment of sFasL levels in fibroblasts’ cell cultures, the fibroblasts isolated from the fibrotic mouse lung were then co-cultured with Jurkat T-cells. In contrast to bleomycin-treated WT-mouse lung fibroblasts, 70% of MMP-7KO mouse lung myofibroblasts died in the co-culture with T cells (Figure 4B, microscope images, MMP-7KO vs. WT +T cells vs. MMP-7KO or WT). Their cell number decreased from 3 × 10^5^ (Figure 4B, insert with numbers-Ctrl) to only 0.9 × 10^5^, while lung myofibroblasts from WT mice maintained relatively stable cell numbers in the co-culture (from 3 × 10^5^ to 2.8 × 10^5^). There were also higher rates of myofibroblast cell deaths in the MMP-7KO vs. the WT co-cultures (Figure 4C, WT and MMP7-KO myofibroblasts co-cultured with T cells). These results indicate that MMP-7 is one of the main mechanisms in the generation of sFasL and in rendering fibrotic-lung myofibroblasts resistant to cell death. Thus, fibroblasts ‘entered’ into the co-culture with T cells with different ‘starting levels’ of sFasL. MMP7-KO fibroblasts, for example, possessed a negligible capacity to release sFasL (Figure 4A). Thus, MMP7-KO fibroblasts, with a negligible capacity for sFasL release, had an inferior survival starting point compared to WT fibroblasts. Indeed, the increased cell death of MMP7-KO fibroblasts caused an extended decrease in the sFasL levels, which may have further diminished their capacity for cell survival. Of note, the number of WT and MMP-7 KO living cells, before and entering the co-culture, was approximately the same (3 × 10^5^ per well). 

## 4. Discussion

We have previously shown that IPF-lung myofibroblasts resist FasL and T cell-induced cell death by the secretion of sFasL [32]. MMPs are known to cleave the mFasL membrane-bound form to soluble–sFasL [21]. In this study, we show that IPF-, as well as normal-lung fibroblasts retain mFasL and release less sFasL into culture medium following exposure to the broad-spectrum MMP inhibitor batimastat (Figure 1). Moreover, batimastat causes fibrotic-lung myofibroblasts to lose their resistance to cell death (Figure 2). In addition, bleomycin-treated MMP-7KO mice had relatively normal and low sFasL levels in their serum compared to WT mice (Figure 3A, and insert) and in contrast to IPF-patients [45,46], who show increased sFasL levels in their blood stream during fibrosis. Additionally, myofibroblasts from bleomycin-treated MMP-7KO mouse lungs did not release sFasL into culture medium (Figure 3B), and these mouse lung myofibroblasts lost their resistance to T cell-induced cell death in the co-culture compared to lung myofibroblasts isolated from the lungs of bleomycin-treated (day 14) WT counterparts (Figure 4). It is known that treatment with the pan-MMP inhibitor decreases sFasL levels in cultured cells [49]. It can be assumed therefore that batimastat inhibits the FasL cleavage to the soluble form (sFasL) by metalloproteinases such as MMP-7, MMP-9, and MMP-3, leading to the increased susceptibility of fibroblasts to cell death. In addition, MMPs – and MMP-7 in particular – have been shown to cleave a range of biologically active substrates that may promote or exacerbate fibrosis, such as osteopontin [35,36]. As we have discussed in an extended review published by us previously, osteopontin, via its interaction with CD44, is capable of affecting cell migration [50]. In particular, the interaction of CD44 with osteopontin facilitates change in the cell adhesion capacity and rolling attachments for the extracellular matrix [50], and may thus drive fibroblast migratory capacities, as is the case in fibrosis. Moreover, osteopontin is capable of upregulating the extracellular matrix proteins such as collagen 1 and the matrix regulator TIMP1 [51].

However, the ability of MMP-7 to cleave FasL [33] and the capacity of sFasL to shield myofibroblasts from immune surveillance [32] are important factors to consider in relation to IPF that are nearly universally overlooked in the discussion of the role of MMP-7 in IPF. This study brings to light this role of MMPs in the disease. In fact, to our knowledge, this study is the first to address MMP7′s role in IPF-lung myofibroblast survival and its implications for the evolution of fibrosis.

FasL is a type II transmembrane protein belonging to the TNF family, originally identified for its ability to induce the rapid cell death of Fas^+^ target cell populations [52,53]. Similar to other members of the TNF family, mFasL can be cleaved from the cell surface by membrane MMPs to release sFasL [54,55,56]. The two forms of FasL exhibit significant functional differences [18,20,57]. Although mFasL is both proapoptotic and proinflammatory, sFasL fails to induce cell death or inflammation and can even block the proinflammatory effects of mFasL [18,20]. 

Physiological fibrosis is a normal process whereby damaged tissue undergoes repair by healthy scar formation, followed by scar resolution and the return to normal organ function. Fas ligand (FasL) is an important molecule involved in maintaining immune privilege in normal and fibrotic lungs [38]. Pathological fibrosis, however, is characterized by the myofibroblast evasion of immune surveillance, leading to unremitting myofibroblast accumulation and scarring [38]. This causes irreparable damage to function, with progression to organ failure. It has been shown that patients with IPF possess a marked elevation in the level of sFasL [46], which indicates that it may be important for the pathogenesis of the disease. Moreover, it was shown by us that sFasL plays a critical role in myofibroblast resistance to T-cell-induced cell death during fibrosis [32].

FasL can be cleaved by metalloproteinases such as MMP-7, ADAM10, and MMP-3 in various cell types and under various conditions, such as osteoblast apoptosis, T-cell activation, and the acquisition of chemoresistance by cancer cells. Moreover, MMP-7, an enzyme involved in the cleavage of the Fas ligand, has been shown to play a significant role in various pathological processes, including cancer metastasis, fibrosis, and more. The assessment of MMP-7 activity is considered to be clinically important for patients with IPF, chronic obstructive pulmonary disease (COPD), small cell lung cancer, adenocarcinoma, and squamous cell carcinoma [25,27,29,30,58,59]. The role attributed to MMP-7 in IPF has related primarily to its importance in the formation and remodeling of the extracellular matrix [51]. However, information on the role of MMPs in the cleavage of mFasL on myofibroblasts, particularly those contributing to lung scarring during pulmonary fibrosis, is lacking. 

**Figure 5:**
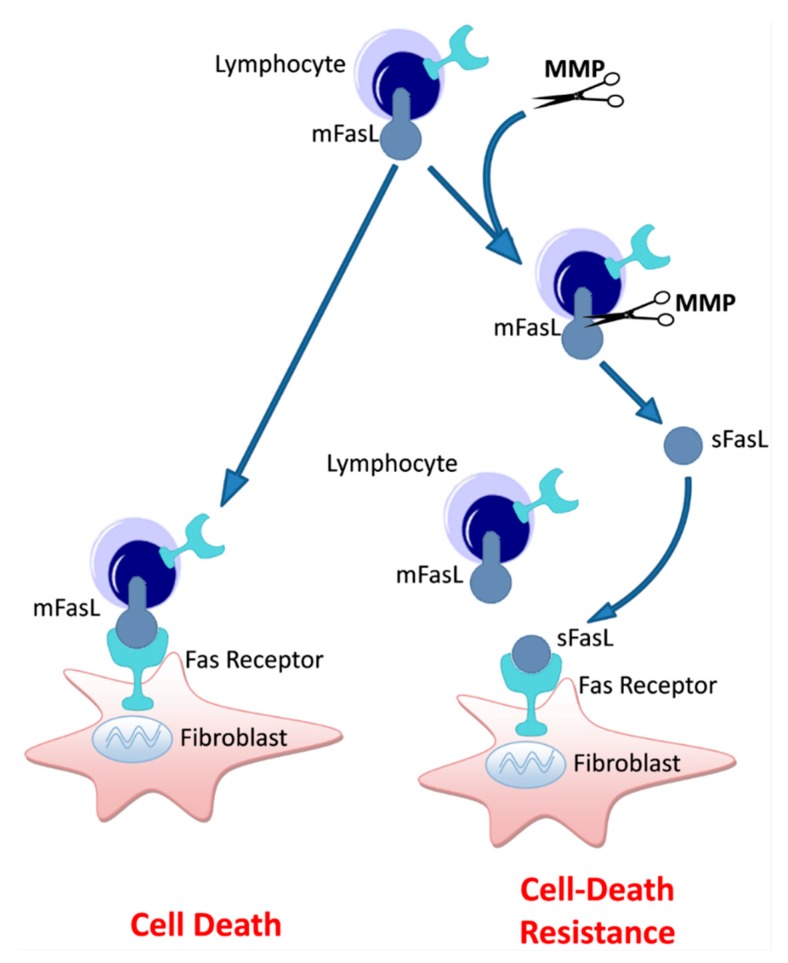
Simplified scheme of the proposed model of sFasL, MMP-mediated, immune cell-induced myofibroblast death regulation during lung fibrosis. Lymphocytes are known to express mFasL that enables T cell-induced myofibroblast cell death with resolution of fibrosis. In fibrotic-lung myofibroblasts, in the presence of MMPs, high levels of sFasL are detected due to mFasL cleavage. High sFasL in the milieu competes with mFasL and limits T cell-induced cell death. sFasL secreted by fibrotic lung fibroblasts is an additional mechanism of their resistance to cell death and their uninterrupted accumulation in lung fibrosis.

MMPs are known to cleave secreted proteins such as collagen and elastin fibers of the extracellular matrix [60], are essential for extracellular matrix remodeling, wound healing, and angiogenesis, and some of them, including MMP-1 and MMP-9, have been implicated in the pathogenesis of IPF [23,51,61,62,63] and other diseases [34]. MMP-7 expression is elevated in both human IPF and murine models of fibrosis, while MMP-7 knockout mice have attenuated fibrotic reactions [23,24]. Nevertheless, there are studies showing that sFasL can be converted from the membrane-bound form by MMPs [21]. Moreover, the importance of sFasL to IPF fibroblast survival and immune avoidance has been demonstrated in our previous work [32]. This supports the understanding of the role of MMPs, and MMP-7 in particular, in pulmonary fibrosis as extending beyond functions such as extracellular matrix remodeling. In our context, human fibrotic lung myofibroblasts release sFasL. Thus, in human fibrotic lung myofibroblasts, the inhibition of MMPs suppressed sFasL secretion and increased their cell death, as depicted in the schematic summary in Figure 5.

To conclude, the present study shows that myofibroblasts from IPF lungs resist cell death by a mechanism involving cleavage of mFasL to sFasL by MMP-7. To the best of our knowledge, the present study is the first to establish the link between MMP-7 activity and the progression of fibrosis via cleavage of FasL and consequent fibroblast resistance to cell death by immune cells. As has been shown, levels of sFasL in the blood correlate with disease progression in IPF patients [46]. This study suggests that MMP-7 knockout mice resist bleomycin-induced fibrosis due at least in part to the decrease in sFasL levels in their blood. The key role that sFasL plays in the pathogenesis of IPF should be explored further, and downregulation of sFasL can serve as a therapeutic strategy in IPF.
